# Identification of Rice *Koji* Extract Components that Increase β-Glucocerebrosidase Levels in Human Epidermal Keratinocytes

**DOI:** 10.3390/foods7060094

**Published:** 2018-06-18

**Authors:** Kazuhisa Maeda, Yuuka Ogino, Ayano Nakamura, Keiji Nakata, Manabu Kitagawa, Seiki Ito

**Affiliations:** 1 Bionics Program, Tokyo University of Technology Graduate School, 1404-1 Katakuramachi, Hachioji City, Tokyo 192-0982, Japan; 2 School of Bioscience and Biotechnology, Tokyo University of Technology, 1404-1 Katakuramachi, Hachioji City, Tokyo 192-0982, Japan; yk.lax6054@gmail.com; 3 MARUKOME Co., Ltd., 883 Amori, Nagano-shi, Nagano 380-0943, Japan; ayano_nakamura@marukome.co.jp (A.N.); keiji_nakata@marukome.co.jp (K.N.); manabu_kitagawa@marukome.co.jp (M.K.); seiki_itou@marukome.co.jp (S.I.)

**Keywords:** *Aspergillus oryzae*, rice *koji*, phosphatidic acid, lysophosphatidic acid, β-glucocerebrosidase

## Abstract

Rice miso contains many ingredients derived from rice *koji* and has been a valuable source of nutrition since ancient times. We found that the consumption of rice miso led to improvements in the moisture content of cheek stratum corneum, skin viscoelasticity, and skin texture. Further, rice miso extract was found to increase the mRNA expression and activity of β-glucocerebrosidase (β-GCase), an enzyme involved in ceramide synthesis in the stratum corneum, in cultures. In this study, we identified the lipid-derived components of rice *koji* that increase the β-GCase activity in cultured human epidermal keratinocytes. The methanol fraction of rice *koji* extract induced an increase in the mRNA expression and activity of β-GCase in keratinocytes. The active fraction of rice *koji* was found to contain phosphatidic acid (PA) and lysophosphatidic acid (LPA). The total PA concentration in rice *koji* was 973.9 ng/mg dry weight, which was 17.5 times higher than that in steamed rice. Among the molecular species, PA_18:2/18:2 was the most frequently found. The total LPA concentration in rice *koji* was 29.6 ng/mg dry weight, and 2-LPA_18:2 was the most frequently found LPA. Since PA and LPA increase the mRNA expression and activity of β-GCase in keratinocytes, they are thought to be the active ingredients in rice *koji* that increase the β-GCase levels in human epidermal keratinocytes.

## 1. Introduction

Rice *koji*, which is a solid-state culture of *koji* molds (*Aspergilus oryzae*) in rice, is used as a raw material for rice miso. Rice miso contains many ingredients derived from rice *koji* and has been a valuable source of nutrition since ancient times. We found that ingesting rice miso improved the moisture content in cheek stratum corneum, skin viscoelasticity, and texture [1]. Furthermore, the effect of miso extract on ceramide synthesis in epidermal keratinocytes was examined in cultured cells, and the mRNA expression and activity of glucocerebrosidase (β-GCase) in these cells increased when the cells were treated with miso extract [1]. From these results, it was hypothesized that the moisture level of the skin improves by everyday consumption of miso soup made from rice miso and that this may occur via increasing the ceramide content of the stratum corneum [1]. *A. oryzae* produces and secretes many types of enzymes, including α-amylase, which degrades starch; glucoamylase; transglucosidase; acid protease, which degrades protein; and acidic carboxypeptidase [2,3], as it grows. In addition, rice *koji* contains various nutrients and supplies the necessary nutrients for yeast and lactic acid bacteria. These nutrients are especially rich in vitamins of the B group and include molybdenum; biotin; vitamins B1, B2, and B6; folic acid; pantothenic acid; and niacin [4]. Furthermore, mono- and diacylglycerol are converted to free fatty acids by the lipid-decomposing enzymes of rice *koji* [5], and other studies have suggested that the free fatty acid content in miso contributes to its antimutagenic activities [6]. The main constituent fatty acids of *A. oryzae* are lignoceric acid and, particularly, 2-oxy lignoceric acid and 2,3-dioxyl lignoceric acid, with phytosphingosine reported as the major long-chain constituent [7]. Rice bran malt contains a bifidobacterial growth-promoting substance and glucosylceramide, which improve intestinal bacterial flora in mice [8,9]. It has also been reported that *A. oryzae* exhibits antioxidant activity [10] and produces multiple cathepsin B-inhibiting components [11]. Rice *koji* is used for most Japanese fermented foods, including miso, soy sauce, amazake, Japanese sake, and malt vinegar. Rice *koji* and sake lees have also been used as cosmetics and skin moisturizing agents for many years. In this paper, we report on the lipid-derived components of rice *koji* that increase β-GCase levels in cultured human epidermal keratinocytes.

## 2. Materials and Methods

### 2.1. Materials

Human epidermal keratinocytes used for a three-dimensional epidermis model were purchased from the Japan Tissue Engineering Co. Ltd. (Tokyo, Japan). Rice *koji,* which is a solid-state culture of *koji* molds (*A. oryzae*) in rice, was obtained from Marukome Co., Ltd. (Nagano, Japan). Reagents not described in the text were obtained from Wako Pure Chemical Industries, Ltd. (Osaka, Japan).

### 2.2. Preparation of Rice Koji Extract

Five hundred grams of fermented rice *koji* produced by an *A. oryzae* addition to steamed rice was extracted using 3 L of chloroform/methanol (2:1). The extract was filtered and concentrated under reduced pressure on a rotary evaporator. The concentrated extract was fractionated sequentially with 3 L of chloroform, 3 L of acetone, and 3 L of methanol using a silica gel column (5 cm × 35 cm), and the fractionated solution was recovered. After concentrating this solution under reduced pressure with an evaporator, the weight of the sample was measured, and each sample was adjusted to a 5 mg/mL concentration by the addition of ethanol.

### 2.3. Effects of Fractions of Rice Koji Extracts on Cell Proliferation and β-GCase Activity in Cultured Human Keratinocytes

Human epidermal keratinocytes were seeded in a 96-well plate at a density of 1 × 10^4^ cells/well, cultured in Dulbecco’s modified Eagle’s medium containing 10% fetal bovine serum (FBS-DMEM) for 24 h and exchanged into 99 μL of 2% FBS-DMEM. Subsequently, 1 μL each of the chloroform fraction, acetone fraction, and methanol fraction of the chloroform/methanol (2:1) extract of rice *koji* was added (*n* = 4). Then, the cells were cultured in a CO_2_ incubator for three days. After culturing, 10 μL of Cell Counting Kit-8 solution (Dojindo Laboratories, Inc., Kumamoto, Japan) was added to each well, and after 2 h, the absorbance (at 450 nm) was measured with a microplate reader (Multi-Detection Microplate Powerscan HT; BioTek, Winooski, VT, USA) to obtain the number of cells. In this method, the tetrazolium salt WST-8 (2-(2-methoxy-4-nitrophenyl)-3-(4-nitrophenyl)-5-(2,4-disulfophenyl)-2H-tetrazolium, monosodium salt) is reduced by intracellular dehydrogenases, and the absorbance at 450 nm of the generated water-soluble formazan is measured to determine the number of living cells. The number of cells and the amount of formazan produced are linearly proportional.

β-GCase activity was determined using 4-methylumbelliferyl β-d-glucopyranoside [1]. Human keratinocytes cultured for three days in the 96-well plate were washed twice with PBS, and 30 μL of 0.01 mol/L acetate buffer (pH = 5.0) and 40 μL of a 5 mmol/L 4-methylumbelliferyl β-d-glucopyranoside solution were added. After a 1 h reaction in an incubator, 50 μL of a 0.1 mol/L glycine sodium hydrate buffer solution (pH = 10.7) was added to determine the fluorescence using the microplate reader. The measurement was performed at an excitation wavelength of 360 nm and an emission wavelength of 460 nm.

### 2.4. Effects of Fractions from Silica Gel Column Re-Fractionated Rice Koji Extracts on Cell Proliferation and β-GCase Activity in Cultured Human Keratinocytes

Methanol fractions ①–⑦ of rice *koji* extract were separately dried and then dissolved in 6 mL of chloroform. The entire volume of dissolved lipid solution was added to 1 g of silica gel in a column (Strata SI-1, Phenomenex Inc., Torrance, CA, USA) equilibrated with chloroform and then eluted with 40 mL of chloroform/methanol (2:1). Subsequently, phospholipids other than lysophospholipids were eluted with 40 mL of chloroform/methanol (1:1). Each fraction was weighed after being dried under reduced pressure. Then, lysophospholipids were obtained with 40 mL of methanol. Each phospholipid or lysophospholipid fraction was separated and weighed after being dried under reduced pressure. Their concentrations were adjusted to 5 mg/mL. Human keratinocytes with these fractions at a concentration of 50 μg/mL were cultured in 2% FBS-DMEM for three days, and the number of cells and β-GCase activity were measured in the same manner as in Section 2.3.

### 2.5. TLC Analysis of Components in Rice Koji That Affect β-GCase Activity

Methanol fractions ②–⑥ (chloroform/methanol 1:1 fractionation, 10 mg/mL); methanol fractions ④–⑥ (methanol re-fractionation, 10 mg/mL); 1,2-di-(cis-9-octadecenoyl)-*sn*-glycerin 3-phosphate sodium salt (PA_C18:1/C18:1, Sigma-Aldrich Corp., St. Louis, MO, USA; 10 mg/mL), as a standard of phosphatidic acid (PA); and oleoyl-l-α-lysophosphatidic acid sodium salt (1-LPA_C18:1, Sigma-Aldrich Corp., St. Louis, MO, USA; 10 mg/mL), as a standard of lysophosphatidic acid (LPA), were spotted on a thin-layer chromatography (TLC) plate (silica gel 60 TLC aluminum plates, Merck KGaA, Darmstadt, Germany). The plate was developed with chloroform/methanol/water/triethylamine (30:35:7:35 (*v*/*v*)). The developed plate was sprayed with molybdenum blue reagent and left at room temperature till turning a blue color. Phospholipids in the sample were estimated from the Rf value of the obtained spots. The spot density was analyzed by ImageJ. The method of preparing the molybdenum blue reagent (coloring reagent) was as follows. Solution A was prepared by adding 4.01 g of molybdenum trioxide (MoO_3_) to 100 mL of 25 mol/L H_2_SO_4_, which was then boiled gently until the solid dissolved. Solution B was prepared by adding 0.18 g of powdered molybdenum (Mo) to 50 mL of solution A, which was then boiled gently for 15 min and allowed to cool. Equal amounts of the supernatants were mixed. After diluting three times with water, an equal volume of ethanol was added, and the solution was placed in a nebulizer.

### 2.6. Effects of PA_C18:1/C18:1, PA_C16:0/C18:1, and 1-LPA_C18:1 on Cell Proliferation and β-GCase Activity in Cultured Human Keratinocytes

PA_C18:1/C18:1,1-palmitoyl-2-oleoyl-*sn*-glycero-3-phosphatidic acid (PA_C16:0/C18:1) from the Cayman Chemical Company, Ann Arbor, MI, USA, and 1-LPA_C18:1 were used. Their concentrations were adjusted to 10 mg/mL, 1 mg/mL, 0.1 mg/mL, and 0.01 mg/mL with ethanol, respectively. The effect of PA_C18:1/C18:1, PA_C16:0/C18:1, and 1-LPA_C18:1 on the number of cells and β-GCase activity in cultured human keratinocytes was examined as described in Section 2.3.

### 2.7. Effects of the PA and LPA Fractions of Rice Koji on TEWL on a Three-Dimensional Human Epidermis Model

An agar plate was placed on a warmer set at 32 °C in a room with a temperature of 22 °C and a humidity of 40%, and a three-dimensional human epidermis model, which had been cultured for four days in the presence or absence of the PA fraction ⑤ or LPA fraction ⑤ of rice *koji* (10 μg/mL, 100 μg/mL in a solvent of ethanol:water (1:99)), was placed on the agar plate inlay. The transepidermal water loss (TEWL) was measured with a Tewameter TM 210 (Courage and Khazaka Electronic GmbH, Köln, Germany) using a probe for 24-well culture inserts.

### 2.8. Effects of PA and LPA on TEWL in a Three-Dimensional Human Epidermis Model

An agar plate was placed on a warmer set at 32 °C in a room with a temperature of 22 °C and a humidity of 40%, and a three-dimensional human epidermis model, which had been cultured for four days in the presence or absence of PA_C18:1/C18:1, PA_C16:0/C18:1, or 1-LPA_C18:1 (10 μ/mL, 100 μg/mL in a solvent of ethanol:water (1:99)), was placed on the agar plate inlay. The TEWL was measured with the Tewameter TM210 using the probe for 24-well culture inserts [12].

### 2.9. Effects of the PA and LPA Fractions of Rice Koji, PA_C18:1/C18:1, PA_C16:0/C18:1, and 1-LPA_C18:1 on the Amount of β-GCase mRNA in the Three-Dimensional Human Epidermis Model

We investigated the amount of β-GCase mRNA involved in ceramide synthesis using a human epidermal model cultured for four days in the absence or presence of the PA fraction of rice *koji*, the LPA fraction, PA_C18:1/C18:1, PA_C16:0/C18:1, or 1-LPA_C18:1 (10 μg/mL, 100 μg/mL in a solvent of ethanol:water (1:99)). RNA extraction was performed using the RNeasy Protect Mini Kit (RNeasy Mini kit, Qiagen GmbH, Hilden, Germany) according to the manufacturer’s instructions. cDNA conversion from the extracted RNA was performed using the One Step SYBR^®^ Prime Script™ RT-PCR Kit II (Takara Bio Inc., Shiga, Japan) according to the package insert. The expression level of mRNA was measured using real-time PCR (Applied Biosystems 7900HT, Thermo Fisher Scientific Inc., Waltham, MA, USA). GAPDH was used as a reference gene for normalization. Primers were purchased from Qiagen. Each experiment was performed thrice. In the analysis, a threshold cycle Ct was obtained, the difference between the Ct value of the housekeeping gene and the Ct value of the target gene (ΔCt: (target gene Ct) − (housekeeping gene Ct)) was obtained, and the ratio of gene expression was calculated from the difference in ΔCt between samples (ΔΔCt).

### 2.10. Measurement of the PA and LPA Contents in Steamed Rice and Rice Koji

Five hundred microliters of methanol and 500 μL of chloroform were added to 20 mg of lyophilized powder of steamed rice and rice *koji*, and after shaking and centrifugation, 100 μL of supernatant was collected. Then, 100 μL of a methanol solution of a PA measurement internal standard (PA_16:0 D 31/18: 1 (500 ng/mL)) was added to this supernatant sample to yield an analytical sample for PA measurement. In addition, 300 μL of the supernatant was taken, and 150 μL of the methanol solution of internal standard 1-LPA_17:0, 2-LPA_17:0 (50 ng/mL) was added to prepare an analytical sample for LPA measurement. Liquid chromatography (LC) and mass spectrometry (MS) conditions are shown below.

LC conditions
DeviceUltiMate 3000 BioRS (Thermo Fisher Scientific Inc.)ColumnL-column 2 ODS metal-free column (2 mm ID × 150 mm, 3 μm, Chemicals Evaluation and Research Institute, Tokyo, Japan)Flow rate0.2 mL/minColumn temperature40 °CSampler temperature5 °CInjection volume3 μLMS conditions
Device3200 QTRAP (AB Sciex Pte Ltd. Framingham, MA, USA)Ionization methodElectrospray ionizationMeasurement modeSRM (negative)

The analysis software Analyst 1.6.1 (AB Sciex) was used for peak detection. The detection limit was set to a signal noise ratio (S/N) of 3. The area value of the detected peak was divided by the peak area value of the internal standard (PA_16:0 D 31/18:1) to calculate the ratio. The approximate concentration of each molecular species in the sample was calculated using the obtained peak area ratio. The coefficient of variation (C.V. %) for five measurements of the peak area ratio (analysis sample/internal standard) of each molecular species was 6.9 ± 2.5 (mean ± standard deviation).

### 2.11. Measurement of the Relative LPA Contents in Steamed Rice and Rice Koji

To prepare the lysophospholipid fraction, 15 mL of chloroform/methanol (2:1) was added to 5 g of each of the three types of steamed rice and rice *koji*, and the mixtures were stirred and extracted at room temperature for 1 h. They were divided into soluble and solid components by filtration, and distilled water was added while stirring to make a chloroform/methanol/water solution (8:4:3 volume ratio). The sample was left undisturbed, and the lower layer (chloroform layer, total lipid fraction) was collected. The total amount of collected lipids was added to a silica gel column (Strata SI-1 100 mg (Phenomenex Inc.), gel volume 1 mL) equilibrated with chloroform; neutral lipids were then eluted using 10 mL of chloroform, and glycolipids were eluted using 40 mL of acetone. Finally, a total phospholipid fraction was obtained with 10 mL of methanol. Total phospholipids were dried and then dissolved in 1 mL of chloroform. The dissolved phospholipid solution was added in its entirety to a silica gel column (Strata SI-1 100 mg, Phenomenex Inc.) equilibrated with chloroform, and phospholipids other than lysophospholipids were eluted with 10 mL of chloroform/methanol (1:1). Subsequently, a lysophospholipid fraction was obtained with 10 mL of methanol. This fraction was dried under reduced pressure and dissolved in 100 μL of 10% Triton X-100-isopropanol. For a determination of LPA concentration, the lysophospholipid fraction was methyl-esterified using a fatty acid methyl esterification kit (Nacalai Tesque, Inc., Kyoto, Japan), and the products were analyzed by GC-flame ionization detection (FID) (TC-WAX, GL Sciences Inc., Tokyo, Japan). 1-LPA_C18:1 (Tocris Bioscience, Bristol, UK) was used as a standard. The peak area ratio of each molecular species with a C.V. below 15% for three measurements was adopted.

### 2.12. Statistical Analysis

Statistical analysis was performed using t-tests run with MS Excel. Differences with a *p* value less than 5% were statistically significant (^∗^ *p* < 0.05, ^∗∗^ *p* < 0.01, ^∗∗∗^ *p* < 0.001), and there was no significant difference (ns) above 5%. In addition, when the ratio was greater than 5% but less than 10%, it was considered to be exhibiting a tendency (^+^ *p* < 0.1).

## 3. Results

### 3.1. Effects of Rice Koji Extract Fractions on Cell Proliferation and β-GCase Activity in Cultured Human Keratinocytes

**Figure 1:**
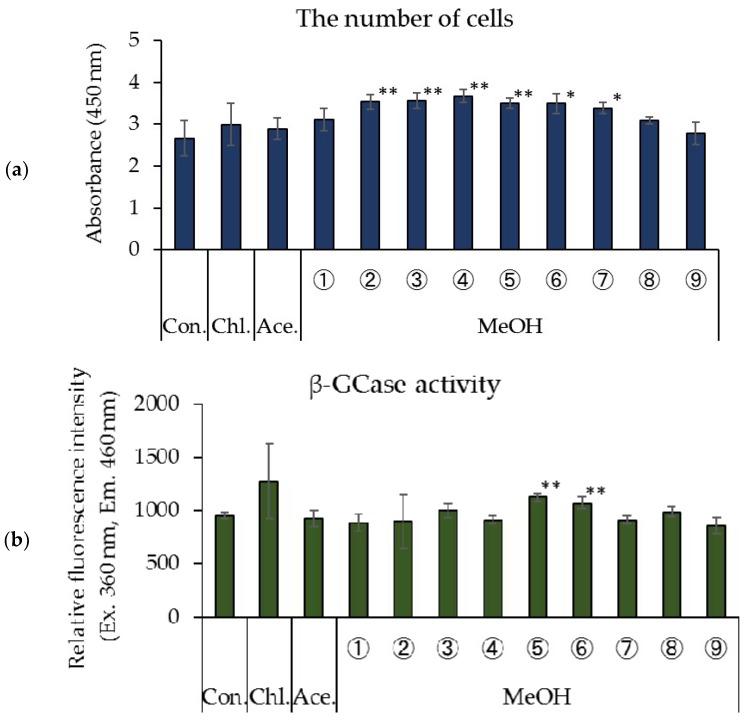
Effects of fractions of rice *koji* extracts on cell proliferation and β-GCase activity in cultured human keratinocytes. (**a**) The number of cells, (**b**) β-GCase activity, *n* = 4, mean ± standard deviation (S.D.), ^+^ *p* < 0.1 vs. control, ^∗^ *p* < 0.05 vs. control, and ^∗∗^ *p* < 0.01 vs. control.

Figure 1 shows the effects of the chloroform, acetone, and methanol fractions of rice *koji* extract on the number of cells and β-GCase activity in cultured human keratinocytes. Significantly more cells were observed in treatments with methanol fractions ②–⑦ of the rice *koji* extract than in the control (Figure 1a). In addition, a significant enhancement in β-GCase activity was confirmed in cells treated with methanol fractions ⑤ and ⑥ of the rice *koji* extract (Figure 1b). The chloroform and acetone fractions of rice *koji* extract had no such effect.

### 3.2. Effects of Fractions from Silica Gel Column Re-Fractionated Rice Koji Extracts on Cell Proliferation and β-GCase Activity in Cultured Human Keratinocytes

**Figure 2:**
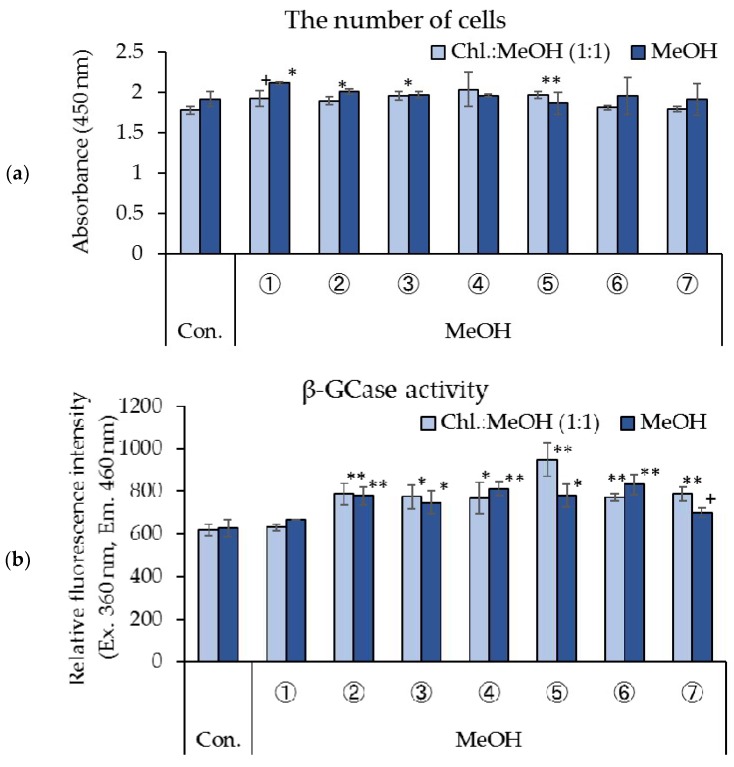
Effects of fractions from silica gel column re-fractionated rice *koji* extract on cell proliferation and β-GCase activity in cultured human keratinocytes. (**a**) The number of cells, (**b**) β-GCase activity, *n* = 4, mean ± S.D., ^+^ *p* < 0.1 vs. control, ^∗^ *p* < 0.05 vs. control, and ^∗∗^ *p* < 0.01 vs. control.

Figure 2 shows the effect of silica gel column re-fractionated rice *koji* extracts on the number of cells and β-GCase activity in cultured human keratinocytes. A significant increase in the number of cells was confirmed in treatments with silica column fractions ②, ③, and ⑤ (chloroform/methanol 1:1 re-fractionation) of the methanol fractions of rice *koji* extract, and a tendency to promote cell proliferation was observed in fraction ① (Figure 2a). In addition, a significant increase in the number of cells was observed in treatments with silica gel column fraction ① (methanol re-fractionation) of the methanol fractions of rice *koji* extract (Figure 2a). A significant increase in β-GCase activity was observed in cells treated with fractions ②–⑦ of the silica gel column fractionations (chloroform/methanol 1:1 re-fractionation) from the methanol fractions of rice *koji* extract (Figure 2b). In addition, a significant enhancement in β-GCase activity was confirmed in cells treated with silica gel column fractions ②–⑥ (methanol re-fractionation) of the methanol fractions of rice *koji* extract, and a tendency to promote β-GCase activity was observed in fraction ⑦ (Figure 2b).

### 3.3. TLC Analysis of Components of Rice Koji Extract That Induce β-GCase Activity

**Figure 3:**
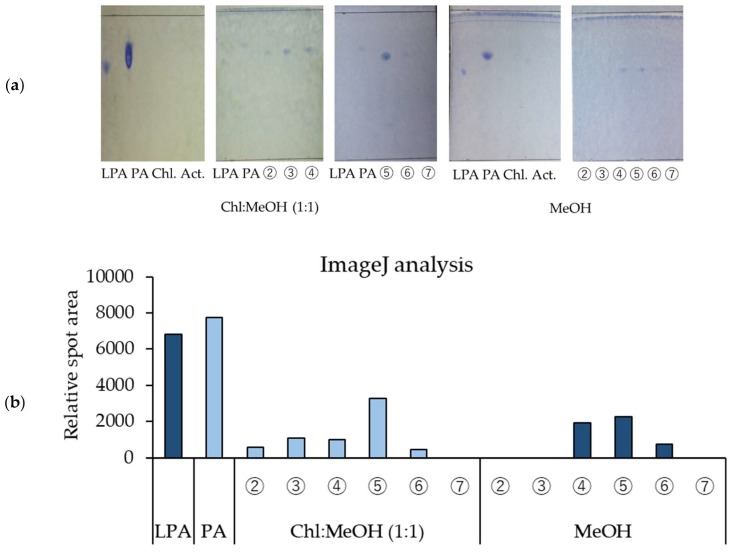
TLC (thin-layer chromatography) analysis of components in rice *koji* extract that increase β-GCase activity. (**a**) TLC image, (**b**) analysis of spot density with ImageJ. LPA: 1-LPA_C18:1, LA: PA_C18:1/C18:1.

In Figure 3a, the TLC images of the chloroform and acetone extracts, methanol fractions ②–⑦ (chloroform/methanol 1:1 re-fractionation of rice *koji* extract) and methanol fractions ②–⑦ (methanol re-fractionation of rice *koji* extract) are shown. The presence of PA in methanol fractions ②–⑥ (chloroform/methanol 1:1 re-fractionation of rice *koji* extract) and LPA in methanol fractions ④–⑥ (methanol re-fractionation of rice *koji* extract) was confirmed. As a result of the analysis of spot density with ImageJ, the presence of PA in methanol fraction ⑤ (chloroform/methanol 1:1 re-fractionation of rice *koji* extract) and of LPA in methanol fractions ④ and ⑤ (methanol refraction of rice *koji* extract) was confirmed in a majority of cases (Figure 3b).

### 3.4. Effects of PA_C18:1/C18:1 and 1-LPA_C18:1 on Cell Proliferation and β-GCase Activity in Cultured Human Keratinocytes

**Figure 4:**
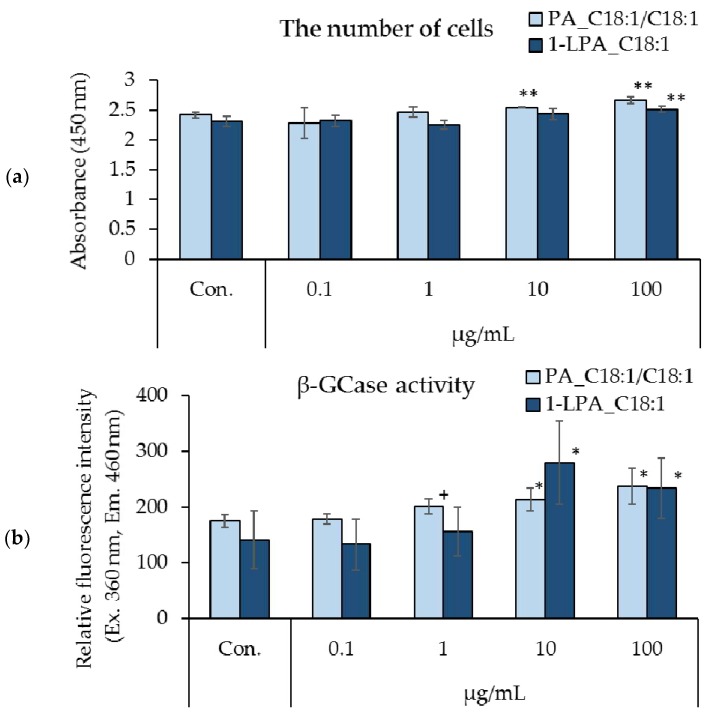
Effects of PA_C18:1/C18:1 and 1-LPA_C18:1 on cell proliferation and β-GCase activity in cultured human keratinocytes. (**a**) Number of cells, (**b**) β-GCase activity; *n* = 4, mean ± S.D., ^+^ *p* < 0.1 vs. control, ^∗^ *p* < 0.05 vs. control, and ^∗∗^ *p* < 0.01 vs. control.

Figure 4 shows the effects of PA_C18:1/C18:1 and 1-LPA_C18:1 on the number of cells and β-GCase activity in cultured human keratinocytes. Treatment with PA_C18:1/C18:1 (10 μg/mL, 100 μg/mL) and 1-LPA_C18:1 (100 μg/mL) resulted in a significant increase in the number of cells (Figure 4a). In addition, a significant enhancement in β-GCase activity was observed in cells treated with either PA_C18:1/C18:1 (10 μg/mL, 100 μg/mL) or 1-LPA_C18:1 (10 μg/mL, 100 μg/mL). Treatment with PA_C18:1/C18:1 (1 μg/mL) also demonstrated a tendency towards increased β-GCase activity (Figure 4b).

### 3.5. Effects of the PA and LPA Fractions of Rice Koji Extract, PA_C18:1/C18:1, PA_C16:0/C18:1, and 1-LPA_C18:1 on the TEWL in the Three-Dimensional Human Epidermal Model

**Figure 5:**
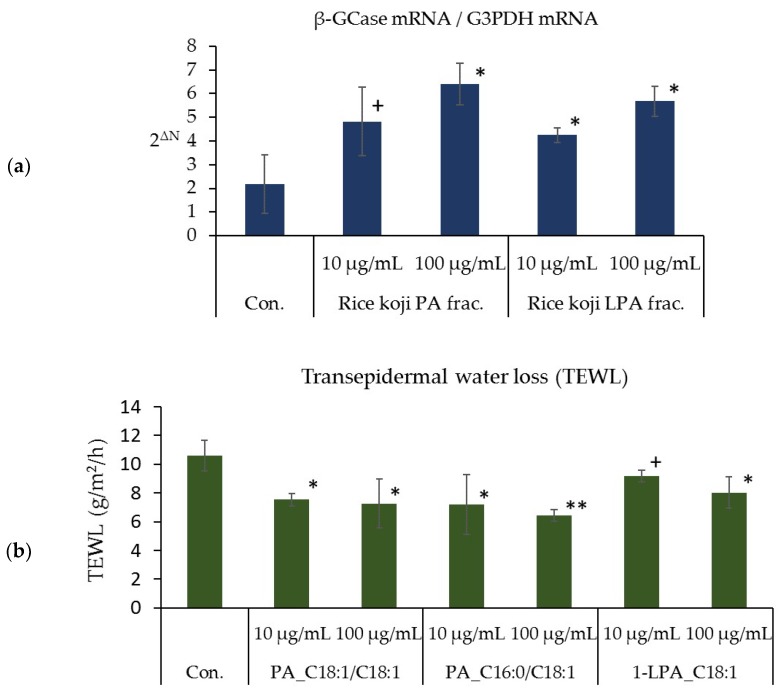
Effects of the PA and LPA fractions of rice *koji* extract, PA_C18:1/C18:1, PA_C16:0/C18:1, and 1-LPA_C18:1 on the TEWL from the three-dimensional epidermal model. (**a**) Effects of the PA and LPA fractions of rice *koji* extract on the TEWL, (**b**) effects of PA_C18:1/C18:1, PA_C16:0/C18:1, and 1-LPA_C18:1 on the TEWL; *n* = 3, mean ± S.D., ^+^ *p* < 0.1 vs. control, ^∗^ *p* < 0.05 vs. control, and ^∗∗^ *p* < 0.01 vs. control.

Figure 5a shows the TEWL, which is the amount of moisture that transpired from the stratum corneum in the three-dimensional human epidermal model cultured for four days in the presence or absence of the PA and LPA fractions of rice *koji* extract. The TEWL from human epidermal models cultured in the presence of methanol fraction ⑤ (chloroform/methanol 1:1 re-fractionation, 10 μg/mL, 100 μg/mL) as the PA fraction and in the presence of methanol fraction ⑤ (methanol re-fractionation, 100 μg/mL) as the LPA fraction was significantly lower than that in the absence of these fractions (control). In addition, the TEWL from human epidermal models cultured in the presence of the LPA fraction (10 μg/mL) had a tendency to be lower than that in the control (Figure 5a).

Furthermore, Figure 5b shows the TEWL from human epidermal models cultured for four days in the presence or absence of either PA or LPA. The TEWL from human epidermal models cultured in the presence of PA_C18:1/C18:1 (10 μg/mL, 100 μg/mL), PA_C16:0/C18:1 (10 μg/mL, 100 μg/mL), and 1-LPA_C18:1 (100 μg/mL) was significantly lower than that in the absence of these fractions (control), and the TEWL in the epidermis model cultured for four days in the presence of 1-LPA_C18:1 (10 μg/mL) showed a tendency to be lower than the TEWL in the control (Figure 5b).

The transpiration of moisture from the stratum corneum was significantly suppressed by PA and LPA.

### 3.6. Effects of the PA and LPA Fractions of Rice Koji Extract, PA_C18:1/C18:1, PA_C16:0/C18:1, and 1-LPA_C18:1 on the Expression Level of β-GCase mRNA in the Three-Dimensional Human Epidermis Model

**Figure 6:**
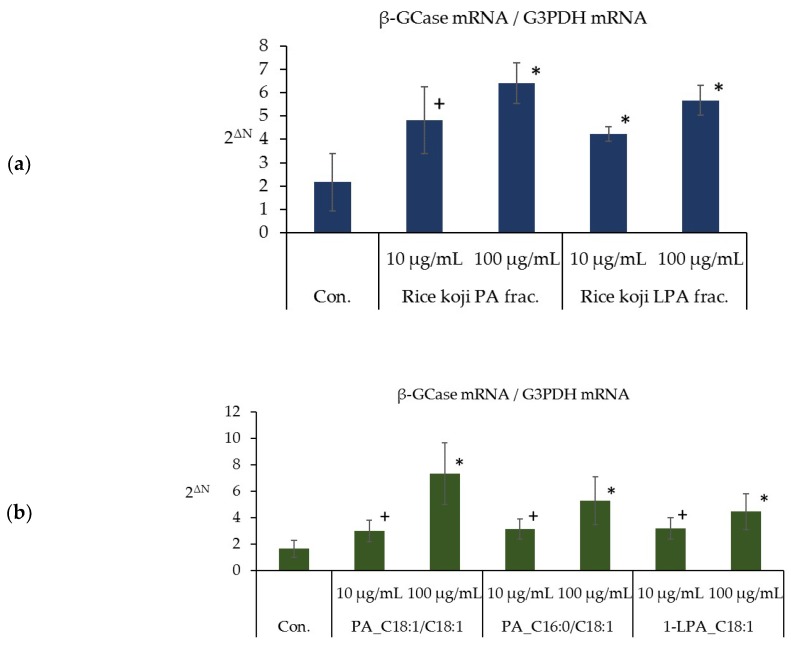
Effects of the PA and LPA fractions of rice *koji* extract, PA_C18:1/C18:1, PA_C16:0/C18:1, and 1-LPA_C18:1 on β-GCase mRNA expression in cultured human keratinocytes. (**a**) Effects of the PA and LPA fractions of rice *koji* extract on the level of β-GCase mRNA, (**b**) effects of PA_C18:1/C18:1, PA_C16:0/C18:1, and 1-LPA_C18:1 on the level of β-GCase mRNA; *n* = 3, mean ± S.D., ^+^ *p* < 0.1 vs. control, ^∗^ *p* < 0.05 vs. control, and ^∗∗^ *p* < 0.01 vs. control.

The level of β-GCase mRNA in the three-dimensional human epidermis models cultured for four days in the presence of either rice *koji* methanol fraction ⑤ (chloroform/methanol 1:1 re-fractionation, PA fraction 100 μg/mL) or methanol fraction ⑤ (methanol re-fractionation, LPA fraction 10 μg/mL, 100 μg/mL) was significantly higher than that in the control (Figure 6a). The mRNA level of β-GCase in the epidermal model cultured for four days in the presence of methanol fraction ⑤ (chloroform/methanol 1:1 re-fractionation, PA fraction 10 μg/mL) tended to be higher than the mRNA level of β-GCase in the control (Figure 6a). Furthermore, the level of β-GCase mRNA in the epidermal models cultured for four days in the presence of PA_C18:1/C18:1 (100 μg/mL), PA_C16:0/C18:1 (100 μg/mL), or 1-LPA_C18:1 (100 μg/mL) was significantly higher than that found in the control epidermal model (Figure 6b). The level of β-GCase mRNA in the models cultured for four days in the presence of PA_C18:1/C18:1 (10 μg/mL), PA_C16:0/C18:1 (10 μg/mL), or 1-LPA_C18:1 (10 μg/mL) tended to be higher than that of the control model (Figure 6b).

### 3.7. Measurement of the PA and LPA Contents in Steamed Rice and Rice Koji

**Figure 7:**
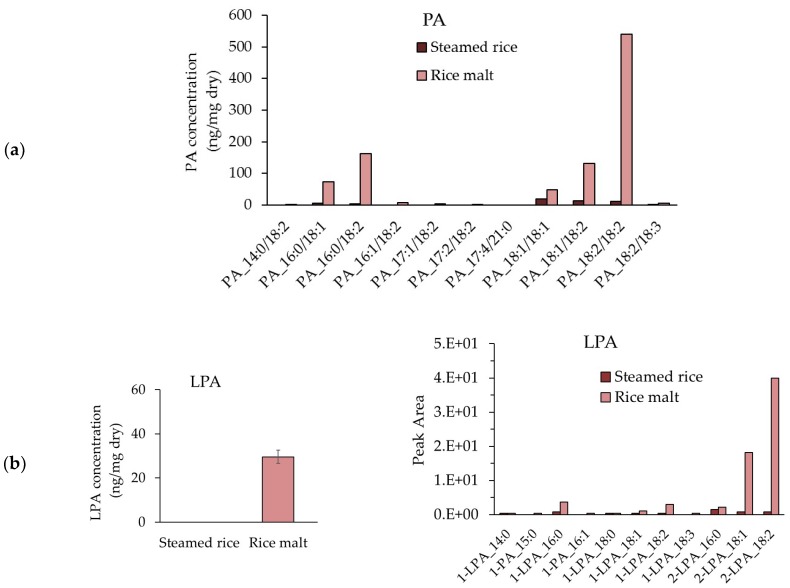
PA and LPA contents in steamed rice and rice *koji*. (**a**) Molecular species content of PA in steamed rice and rice *koji*, (**b**) LPA contents in steamed rice and rice *koji* (left), and molecular species content of LPA in rice *koji* relative to that in steamed rice (right).

The content of PA in each molecular species of steamed rice and rice *koji* was measured, and the results are shown in Figure 7a. The total PA in rice *koji* was 973.9 ng/mg dry weight, which was 17.5 times higher than the total PA in steamed rice (55.5 ng/mg of dry weight). PA_18:2/18:2 is the most common molecular species, followed by, in decreasing order, PA_16:0/18:2, PA_18: 1/18:2, and PA _16:0/18:1 (Figure 7a). The LPA contents of steamed rice and rice *koji* are shown in Figure 7b. LPA was not detected in steamed rice, but LPA at a concentration of 29.6 ng/mg dry weight was confirmed in rice *koji*. In addition, the 2-LPA content in rice *koji* was 14 times that of 1-LPA. The 2 -LPA_18:2 species was the most frequently found 2-LPA, followed by 2-LPA_18:1. Little 1-LPA was found.

### 3.8. Measurement of the PC and LPC Contents in Steamed Rice and Rice Koji

**Figure 8:**
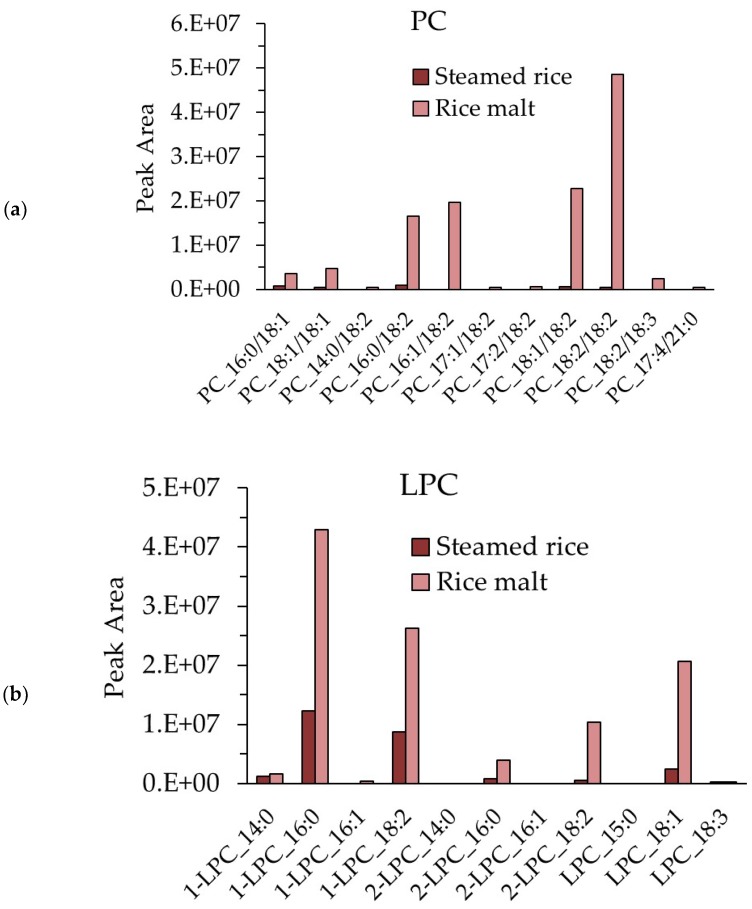
PC and LPC contents in steamed rice and rice *koji*. (**a**) Relative molecular species content of PC in steamed rice and rice *koji* and (**b**) relative molecular species content of LPC in steamed rice and rice *koji.*

The contents of phosphatidylcholine (PC) and lysophosphatidylcholine (LPC) in steamed rice and rice *koji* are shown in Figure 8. Although PC was hardly detected in steamed rice, PC_18:2/18:2 was the most abundant PC in rice *koji*, followed by PC_18:1/18:2 and PC_16:0/18:2 (Figure 8a). Two types of 1-LPC were present in steamed rice: 1-LPC_16:0 and 1–LPC_18:2. Meanwhile, 1-LPC was also present in rice *koji*, and 1-LPC_16:0 and 1-LPC_18:2 were abundant. Both 2-LPC_16:0 and 2-LPC_18:2 were less abundant in rice *koji* than the other types of LPC (Figure 8b).

## 4. Discussion

The stratum corneum intercellular lipids, which extend into the intercellular space of the stratum corneum (extracellular stratum corneum lipids), act as a barrier between the epidermis and the air of the outside world and are critical to preventing the transpiration of moisture from inside the body and moisture ingression from outside the body. When this barrier is damaged, excessive evaporation occurs, and the stratum corneum becomes dehydrated, while further deterioration causes allergic reactions in the skin, such as rough skin and atopic dermatitis [13,14,15]. The intercellular lipids consist of ceramides (37%), cholesterols (32%), long-chain fatty acids (16%), and cholesterol esters (15%) and form a multilayered lamellar lipid structure [16]. Among these components, ceramides are particularly important in the stratum corneum and are present in amounts 30 times or greater in the stratum corneum than in other organs. The stratum corneum ceramides are characterized not only by their large quantity, but also by the presence of various kinds of molecules [17,18]. Acylceramide, which is important for skin barrier function, is produced by β-GCase from acylglucosylceramide [19,20]. In addition, some ceramides are produced from both glucosylceramide and sphingomyelin [21,22].

We showed that the mRNA expression and activity of β-GCase, which is an enzyme involved in ceramide synthesis in the stratum corneum, increases with exposure to rice miso extract [1]. The levels of phytosphingosine- and sphingosine-type ceramides increase after such exposure [1]. During the fermentation of miso, various functional substances are produced by the enzymatic action of various fermenting microorganisms. For example, miso contains functional substances, such as essential fatty acids (linoleic acid and linolenic acid), and acylphosphatidic acid is produced by *Rhizopus oryzae* [23]. In this study, to investigate whether ceramide synthesis is involved in the skin improvement effects of rice *koji*, we examined the effects of the lipid component of rice *koji* on the mRNA level and activity of β-GCase in cultured keratinocytes. We found that both the mRNA level and activity of β-GCase were increased in cells treated with a methanol fraction of rice *koji* extract and with PA and LPA. In addition, when a methanol fraction of rice *koji* extract, PA, and LPA were allowed to act on the three-dimensional human epidermal skin model, a decrease in TEWL and increases in β-GCase activity and β-GCase mRNA expression occurred. On the other hand, the chloroform and acetone fractions of rice *koji* extract had no such effect. Rice (*Oryza sativa* L.) contains phospholipase A1 and phospholipase A2 [24], and rice bran oil contains phospholipase D [25,26]. Since steamed rice is used for the production of rice *koji*, these enzymes are thought to be inactivated. On the other hand, *A. oryzae* produces phospholipase A1 [27,28]. Rice bran oil contains phospholipids [29], and rice contains many PCs, which are phospholipids, and LPC_16:0 (3.0 to 4.7 μg/mL) and LPC_18:2 (0.8 to 2.2 μg/mL), which are both lysophospholipids [30].

In our study, PA and LPA were included in rice *koji*, and since their concentrations were higher in rice *koji* than in steamed rice, the source of phospholipids was thought to be the rice. The abundance of 2-LPA was 14 times greater than that of 1-LPA in rice *koji*. The 2-LPA_18:2 species was the most frequently found, followed by 2-LPA_18:1, and the 1-LPA level was low in rice *koji*. From these results, PA was considered to be produced from PC by phospholipase D of *A. oryzae*, and 2-LPA was produced from PA by phospholipase A1. Furthermore, these results indicated that the activity of phospholipase A1 of *A. oryzae* was higher than that of phospholipase A2. However, since the PA content in rice *koji* was 10 times greater than the LPA content, the phospholipase A1 activity of *A. oryzae* was not thought to be high. In addition, 2-LPA may be produced by phospholipase D of *A. oryzae* from the 2-LC produced by phospholipase A1 of *A. oryzae*, but the pathway by which 1-LPA is produced from 1-LC via phospholipase D of *A. oryzae* contributes less. The activity of phospholipase D of orthologous genes has not been verified in *A. oryzae* [31], and further investigating the variations in phospholipase D for each *A. oryzae* strain is necessary.

## 5. Conclusions

The methanol fraction of rice *koji* extract contains PA and LPA, both of which increase the mRNA expression and activity of β-GCase in cultured human epidermal keratinocytes. Therefore, PA and LPA are thought to be among the active ingredients in rice *koji* that increase the β-GCase levels in human epidermal keratinocytes. The total PA in rice *koji* was 973.9 ng/mg dry weight, which was 17.5 times higher than that in the steamed rice used as the source of rice *koji*, and contained several molecular species, of which PA_18:2/18:2 was the most abundant. The total LPA in rice *koji* was 29.6 ng/mg dry weight, and 2-LPA_18:2 was the most abundant. Based on these findings, foods containing rice *koji* (which includes high levels of PA and LPA) can presumably function in improving the moisture content, viscoelasticity, and texture of human skin.
